# Draft Genome Sequences of *Vibrio cholerae* O1 ElTor Strains 2011EL-301 and P-18785, Isolated in Russia

**DOI:** 10.1128/genomeA.00659-13

**Published:** 2013-08-22

**Authors:** Konstantin V. Kuleshov, Sergey O. Vodop’ianov, Mikhail L. Markelov, Vladimir G. Dedkov, Anton V. Kermanov, Vladimir D. Kruglikov, Alexey S. Vodop’ianov, Ruslan V. Pisanov, Alexey B. Mazrukho, German A. Shipulin

**Affiliations:** Federal Budget Institute of Science Central Research Institute for Epidemiology, Moscow, Russiaa; Federal Government Health Institution Rostov-on-Don Plague Control Research Institute, Rostov-on-Don, Russiab; Institute of Occupational Health RAMS, Moscow, Russiac

## Abstract

We report the draft whole-genome sequences of two *Vibrio cholerae* O1 strains, the environmental toxigenic strain 2011EL-301 and the clinical nontoxigenic strain P-18785, both isolated in Russia. Some basic data comparing the two against the GenBank repository are provided.

## GENOME ANNOUNCEMENT

Cholera remains one of the most socially significant and dangerous infectious diseases in the world. Sporadic cases and outbreaks are considered to be emergency situations with a threat of international spread. Next-generation sequencing has recently become a promising approach for effective molecular epidemiological investigations, although a large number of sequenced isolates are required for effective genotyping and comparative analyses (1, 2). Here, we report the first draft whole-genome sequences of two *Vibrio cholerae* O1 ElTor strains. The first strain is environmental toxigenic *V. cholerae* O1 ElTor Inaba 301 (2011EL-301), which was isolated from the Azov Sea near Taganrog city in August 2011. The second strain is the nontoxigenic *V. cholerae* О1 ElTor Ogawa P-18785, which was isolated in 2005 from an asymptomatic carrier from Tajikistan during an outbreak of cholera in the Kamensky district of the Rostov region (3).

Genomic DNA was extracted using the standard protocol of phenol-chloroform extraction (4). Libraries for paired-end sequencing were prepared from 1 to 5 µg of genomic DNA using the TruSeq kit, and genomes were sequenced using the MiSeq platform (Illumina, San Diego, CA). Filtering of raw reads and *de novo* assembly of the genomes were performed using the CLC Genomics Workbench v5.0.1 (CLC bio, Aarhus, Denmark). Gene finding and annotation were achieved using the NCBI Prokaryotic Genome Automatic Annotation Pipeline.

The draft genome of 2011EL-301 was assembled into 164 contigs, with 100× average coverage and an N_50_ of 132 kb. The summed contig length of this assembly is 4,008,237 bp. This genome putatively encodes 3,575 protein-coding sequences and 81 RNAs, 77 of which are annotated as tRNAs. The draft genome of the P-18785 strain was assembled into 159 contigs, with an average coverage of 50× and an N_50_ of 83 kb. The summed contig length of this assembly is 4,008,237 bp. This genome putatively encodes 3,587 protein-coding sequences and 67 RNAs, 63 of which are annotated as tRNAs.

A comparative analysis of virulence-associated mobile elements and genomic islands, like prophages CTXφ and RS1φ, *Vibrio* pathogenic islands 1 and 2 (VPI-1 and VPI-2, respectively), *Vibrio* 7th pandemic islands (VSP-1 and VSP-2), and an integrative and conjugative element belonging to the SXT/R391 (ICE-SXT) family revealed that these regions have high nucleotide sequence similarity to and synteny with the same regions of the recently sequenced genomes of altered *V. cholerae* ElTor strains (2). The draft genome sequence of P-18785 contains only part of the VPI-2 (~14 kb) and the whole VPI-1. The prophages CTXφ and RS1φ, islands VSP-1 and VSP-2, and ICE-SXT were absent. The region that is similar to prophage KSF-1φ, also known as genomic island 19, was detected in the contigs that correspond to the second chromosome (5).

Phylogenetic analysis that was based on single-nucleotide variations (SNVs) located in protein-coding genes orthologous to genes in previously sequenced genomes deposited in GenBank showed a clustering of strain 2011EL-301 with the 7th pandemic isolates from the introduction of cholera cases in the United States from Pakistan between 2005 and 2010 and an outbreak strain of *V. cholerae* in Zimbabwe in 2009 (2). In turn, the strain P-18785 clusters with strains that were isolated from the environment of the Gulf Coast region of the United States.

### Nucleotide sequence accession numbers. 

The whole-genome shotgun project for the 2011EL-301 strain has been deposited at DDBJ/EMBL/GenBank under the accession no. AJFN00000000.2. The whole-genome shotgun project for the P-18785 strain has also been deposited at DDBJ/EMBL/GenBank under the accession no. ANHS00000000.
